# Hemodynamics Modify Collagen Deposition in the Early Embryonic Chicken Heart Outflow Tract

**DOI:** 10.3390/jcdd4040024

**Published:** 2017-12-20

**Authors:** Monique Y. Rennie, Stephanie Stovall, James P. Carson, Michael Danilchik, Kent L. Thornburg, Sandra Rugonyi

**Affiliations:** 1 Knight Cardiovascular Institute, Center for Developmental Health, Oregon Health & Science University, Portland, OR 97239, USA; moniquerennie@gmail.com (M.Y.R.); thornbur@ohsu.edu (K.L.T.); 2 Biomedical Engineering, Oregon Health & Science University, Portland, OR 97239, USA; stovalls@ohsu.edu; 3 Texas Advanced Computing Center, University of Texas, Austin, TX 78758, USA; jcarson@tacc.utexas.edu; 4 Integrative Biosciences, Oregon Health & Science University, Portland, OR 97239, USA; danilchi@ohsu.edu

**Keywords:** cardiac development, mechanotransduction, tissue remodeling, congenital heart disease, heart malformation

## Abstract

Blood flow is critical for normal cardiac development. Hemodynamic stimuli outside of normal ranges can lead to overt cardiac defects, but how early heart tissue remodels in response to altered hemodynamics is poorly understood. This study investigated changes in tissue collagen in response to hemodynamic overload in the chicken embryonic heart outflow tract (OFT) during tubular heart stages (HH18 to HH24, ~24 h). A suture tied around the OFT at HH18 was tightened to constrict the lumen for ~24 h (constriction range at HH24: 15–60%). Expression of fibril collagens I and III and fibril organizing collagens VI and XIV were quantified at the gene and protein levels via qPCR and quantitative immunofluorescence. Collagen I was slightly elevated upstream of the band and in the cushions in banded versus control OFTs. Changes in collagen III were not observed. Collagen VI deposition was elevated downstream of the band, but not overall. Collagen XIV deposition increased throughout the OFT, and strongly correlated to lumen constriction. Interestingly, organization of collagen I fibrils was observed for the tighter banded embryos in regions that also showed increase in collagen XIV deposition, suggesting a potentially key role for collagens I and XIV in the structural adaptation of embryonic heart tissue to hemodynamic overload.

## 1. Introduction

Blood flow is needed for normal heart development, and in its absence cardiac formation is severely impaired [1]. During development, constantly changing hemodynamic stresses, exerted by blood flow on the heart walls, shape the structure of the heart and the composition of cardiac tissues, by modulating developmental programs [2]. Perturbations in hemodynamic stresses (blood pressure, wall shear stress) outside the normal range during early embryonic heart development lead to overt cardiac structural changes, e.g., [3,4,5,6,7], that are similar to human congenital heart defects. Yet, the early events that eventually lead to these cardiac structural malformations are only beginning to be understood.

At early stages of development, the embryonic heart has a tubular structure. The tubular heart is composed of four functional segments connected in series: the primitive atrium, atrioventricular canal, primitive ventricle, and outflow tract (OFT). The OFT connects to the ventricle on one side, and to the aortic sac (which connects the heart to the systemic circulation) on the other. Cardiac walls at these stages have three layers: (i) the myocardium, which is the layer that actively contracts; (ii) the endocardium, a monolayer of endocardial cells in contact with blood flow; and (iii) the cardiac jelly, which separates the myocardium and endocardium, and is formed of extra-cellular matrix (ECM). Endocardial cushions, which are thickenings of the wall initially composed of ECM or cardiac jelly, form in the atrioventricular canal and OFT. Prior to valve formation, cells invade the endocardial cushions gradually increasing cell density. The OFT, also known as the conotruncus, later develops into semilunar valves, pulmonary and aortic trunks, and a portion of the ventricular septum, and is particularly prone to structural defects. Human congenital heart defects such as transposition of the great arteries, truncus arteriosus, tetralogy of Fallot (TOF), double-outlet right ventricle (DORV), and ventricular septal defects (VSD) are known to arise from abnormalities of the OFT [8,9]. Interestingly, these defects are also found in animal models after interventions that alter normal blood flow conditions [3,4,5,6,7]. Perturbed hemodynamic conditions, moreover, affect the cellular density of endocardial cushions, presumably by altering the endothelial-mesenchymal transition (EMT) that occurs in the cushions prior to valve formation [10,11,12,13,14,15]. Little is known, however, about the cardiac tissue remodeling (change in composition) that occurs in response to altered hemodynamic conditions. 

In the overloaded adult heart, increased deposition of fibril collagens type I and III is observed across a range of animal models [16,17] and in humans [18,19]. Collagens I and III provide cardiac tissue with the structural integrity required to withstand large dynamic pressure changes during a lifetime of cardiac cycles, and together they account for more than 90% of adult cardiac collagen content. Collagens I and III have been shown to increase in embryonic vascular dorsal aortic tissue in response to hemodynamic overload [20] and collagen I is dramatically upregulated in the embryonic chicken atrio-ventricular cushion tissue when cultured under conditions of flow compared to no-flow conditions [21]. Deposition of the fibril-associated collagens VI and XIV may be affected by hemodynamic overload in the embryonic heart, as both these collagen types play an important role in fibril organization of the heart and other tissues [22,23,24]. Collagen VI is critical for proper valve formation during heart development [25,26], and collagen XIV has been shown to have an important role in adaptation to hemodynamic load in various other embryonic tissues [22,23]. While not the only components of cardiac tissues and cushions, collagens are important structural components of the developing and mature heart tissues. It is therefore natural to study their role in the response to flow during early cardiac development.

Collagens are a family of ECM proteins characterized by triple helical structures, formed by three α chains or subunits. Fibril collagens, which include collagens type I and III, form a long triple helical protein. Collagen type I is formed by two α1(I) chains and one α2(I) chain; collagen type III is formed by three α1(III) chains. Each collagen subunit is encoded by a different gene, e.g., *COL1A1* and *COL1A2* are the genes that encode the collagen I α1 and α2 subunits, respectively. Collagens can also be classified into a class of fibril-associated collagens with interrupted triple helices (FACIT), which include collagens type VI and XIV. These collagen proteins are typically shorter in length than fibril collagens and their triple helical regions are interrupted by globular domains. The triple helix of collagen type VI is composed of three subunits, α1(VI), α2(VI), and α3(VI); while the triple helix of collagen XIV is composed of three α1(XIV) subunits. In this study, we evaluated changes in tissue protein content and gene expression of collagens type I, III, VI and XIV (see Table 1) in the overloaded embryonic tubular heart OFT.

Cardiac overload was achieved via a well-established hemodynamic intervention during tubular stages of chicken heart development, outflow tract banding [5,27,28]. Our group has shown that placing and tightening a suture (band) around the embryonic heart OFT increases blood pressure throughout the embryonic cardiovascular system, and wall shear rates in the banded region, in a band-tightness dependent manner [29,30]. Physically, the suture around the OFT restricts the motion of the OFT wall and reduces the OFT cross-sectional lumen area. This leads to increased blood pressure and increased blood flow velocity through the band site, as stroke volume is conserved [29]. In this study, outflow tract banding (OTB) [5,27,31] performed at the Hamburger–Hamilton (HH) [32] stage 18 (HH18), with the band left in place for 24 h to stage HH24. Our previous study showed that altering hemodynamic conditions through banding for only 24 h (the time window of the experimental design in this manuscript) leads to congenital heart malformations, with roughly 60% incidence of cardiac defects (VSDs, TOF, and DORV) on embryos with HH18 band tightness between 20 and 45% [7]. The study further showed that cardiac defect incidence and phenotype depend on the level of hemodynamic alteration (band tightness). Moreover, we found that after 24 h of banding, OFT cushion cell density increases with respect to control conditions, and this increase is proportional to band tightness [10]. The increase in endocardial cushion cell density, in turn, contributes to early OFT tissue remodeling as cells secrete ECM components, including collagens. Early remodeling of the OFT during tubular stages (HH18–HH24), therefore, is likely responsible for the later incidence of heart defects.

In this study, OFT collagen composition (collagens I, III, VI and XIV) was evaluated at HH24 under banded and controlled conditions. To assess protein and gene expression, we used histology, and quantitative immunofluorescence; and tissue samples were collected for qPCR. Given the importance of the OFT on later cardiac development and the prevalence of human defects that originate from this cardiac segment, we focused our investigation on the OFT. We quantified global and local collagen content in the three distinct layers of the embryonic OFT wall (myocardium, cardiac jelly, endocardium). We found location-specific increases in collagens VI, and XIV, highlighting the importance of these collagens in cardiac adaptation to hemodynamic load during embryonic development.

## 2. Materials and Methods 

### 2.1. Outflow Tract Banding and Sham Preparation

Fertilized White Leghorn chicken eggs were incubated blunt end up in a rocking incubator for approximately 3 days at 38 °C to HH18 [32]. Embryos were exposed by windowing the blunt end of the shell and removing the inner shell membrane in the region of the embryo. Embryonic HH stage was then verified. The embryonic sac was gently opened and pulled back to expose the heart and a 10-0 nylon suture was passed under the middle of the OFT. In outflow tract banded (OTB) embryos, the suture was tied snugly around the OFT wall at various degrees of constriction. In sham controls, the suture was passed under the OFT and then removed. Shell windows were sealed with saran wrap and the eggs were re-incubated for 24 h to stage HH24, with a subset of embryos (used for qPCR) undergoing additional imaging 2 h after banding or sham procedure.

### 2.2. Outflow Tract Diameter and Band Tightness Calculation

To determine the degree of constriction introduced by outflow tract banding (OTB), the external OFT diameter was measured at the band site and at a comparable site (the middle of the OFT) in control embryos. Diameter measurements were made at different stages (HH18 or HH24) depending on the particular procedure performed on the embryos and imaging modality employed (described later). While some data is reported as a function of OFT band diameter, band tightness was also calculated and reported,

(1) $$Band\;Tightness=1-\;\frac{D_{a}}{D_{b}}$$

where $D_{a}$ is the OFT diameter at the location of the band after the band is placed, and *D_b_* is either the maximum OFT diameter at the location of the band before the band was placed, or an average diameter at approximately the location of the band from control embryos. Note that band tightness computed from images at HH18 is not directly comparable to band tightness calculated from images at HH24. Nevertheless, band tightness measurements give us a quantification of OFT constriction that we used for comparison and to better analyze and understand resulting data.

### 2.3. Blood Flow Velocity Measurements at HH24

OFT hemodynamics and wall dimensions were evaluated in vivo at HH24. To this end, we imaged the heart using ultrasound biomicroscopy (Vevo 2100 with a 40 MHz transducer; Visualsonics, Toronto, ON, Canada) 24 h after banding (*n* = 10) or sham procedure (*n* = 8), at approximately HH24. During ultrasound data acquisition, temperature was monitored and controlled with a heat lamp throughout the imaging session. To ensure good contact between the transducer and the embryonic heart, previously warmed (38 °C) Ringer’s solution was placed on top of the egg window. Data were recorded from embryos with heart rates within previously published norms (range: 180–210 beats per minute); as it was previously found that banding does not affect heart rate [30,33]. OFT external diameters at maximal expansion were measured from B-mode images at the band site, or at an equivalent location in sham controls, using digital calipers and averaging over three cardiac cycles (Vevo software, Visualsonics). Doppler waveforms from within the OFT lumen and at the band region were also obtained, and peak velocity (maximum velocity over the cardiac cycle) was determined from an average of three velocity cycles. Embryos used for this study were discarded after ultrasound data acquisition.

### 2.4. Histology

After 24 h of banding or sham procedure (HH24), 4 control and 4 banded hearts were dissected for histology and fixed in 4% paraformaldehyde at 4 °C. They were subsequently dehydrated in methanol, embedded in paraffin and sectioned (5 μm slice thickness). Longitudinal sections through the OFT (at least 4–5 per heart) were stained with Picrosirius red (#24901, Polysciences, Warrington, PA, USA) per kit instructions. Developing times (optimized in preliminary experiments) and section thickness were kept consistent across slides to facilitate analysis. Images were taken on a Leica DM2000 microscope (40× objective) with a Leica DFC 295 camera using Leica Application Suite software (Leica Microsystems, Wetzlar Germany). We used the obtained Picrosirius stained images for a qualitative (descriptive) indication of changes with banding in the OFT cushions.

### 2.5. Whole Embryo Immunofluorescence

In a separate series of embryos, after 24 h of banding or sham procedure (HH24), embryos were dissected and fixed in 4% paraformaldehyde overnight at 4 °C. Whole embryos were dehydrated in methanol, endogenous peroxidase activity blocked with methanol/DMSO/H_2_O_2_, rehydrated and blocked for 45 min with PBSTM +4% goat sera (Jackson ImmunoResearch Laboratories, Inc., West Grove, PA, USA). Embryos were treated with a primary antibody overnight at 4 °C, extensively washed, and treated with an appropriate secondary fluorescent antibody (Rhodamine Red, Jackson Immunoresearch) overnight at 4 °C, after which they were extensively washed. The following primary antibodies were used for immunostaining: rabbit anti-chicken collagen I (PA1-26147, Pierce Biotechnology, Rockford, IL, USA); mouse anti-chicken collagen III (3B2, Developmental Studies Hybridoma Bank, Iowa City, IA, USA); mouse anti-chicken collagen VI (39-c, Developmental Studies Hybridoma Bank); and rabbit anti-chicken collagen XIV (gift from Patrizio Castagnola, National Institute for Research on Cancer, Genova, Italy). Immunostained embryos were then dehydrated in ethanol and cleared in BABB solution (1:2 benzyl alcohol: benzyl benzoate) before confocal microscopy imaging. For each primary antibody, 8 sham and 8 banded (OTB) embryos were immunostained (and then imaged) in 2 to 4 batches. Although solution concentrations and wash timings were strictly maintained throughout the study, by immunostaining, imaging, and analyzing data in batches we hoped to eliminate the effects of potential variations that could result from minor disparities in these parameters from one batch to another. For each batch of immunostaining, an embryo that received secondary antibody, but not primary antibody, was included in the set. This embryo served as a negative control for the antibody staining.

Confocal 3-D image stacks (z-stacks) were captured from immunostained embryonic hearts using a Zeiss/BioRad Radiance 2100 confocal laser scanning system mounted on a Nikon E800 upright compound microscope (10× 0.45 NA Plan Apo DIC objective). Image stacks were simultaneously collected for innate autofluorescence of the heart and Rhodamine Red secondary antibody at 488 and 543 nm, respectively. Image stacks (10 μm/step) containing between 18–30 images (pixel size 1.36 µm, image size 700 µm^2^) were acquired, spanning the OFT cardiac volume. Iris settings were adjusted to approximately match the z-step distance. For banded embryos, OFT diameter was measured from an image in the stack in which the boundaries of the suture knot were clearly visible using Image J (NIH, Bethesda, MD, USA) and band tightness calculated using Equation (1) as explained before.

Quantitative immunofluorescence relies on ensuring that the number of fluorescent probes, and thus confocal light intensity from the sample, is proportional to the amount of the specific protein targeted (e.g., [34,35]). A step toward achieving proportionality is to ensure that sample preparation procedures (timings, concentrations, number of washes, etc.) are strictly maintained in the study (as described above). Confocal image intensity, however, depends also on microscope and image settings (it is a relative measurement). To allow for quantitative comparisons of fluorescent signals (quantitative immunofluorescence), the following additional steps were taken: (1) Instrument settings were manually adjusted for each batch using a sham control. Settings were then maintained for the batch such that observed signal variation within a batch was due to differences in collagen content, rather than differences in image scan parameters. (2) When setting intensities for the sham control for each batch, laser power was set to slightly below image saturation for the most intensely stained regions (often the extraembryonic membrane). As this was done for each batch using a control embryo, intensities between batches were assumed comparable (control embryos showed little variation). (3) A negative control embryo, which was only stained with the secondary but not the primary antibody, was also included as part of the batch as a background intensity control. While additional factors may still influence intensity (e.g., sample bleaching, fluorescence sensitivity), and thus add noise to our data, we strived to keep all variables the same to ensure consistency.

To assess organization of collagen I fibrils, a separate series of collagen I immunostained embryos (*n* = 5/group) was imaged at higher resolution (pixel size 0.69 µm, image size 700 µm^2^) on a Zeiss LSM 780 confocal microscope (20 × 0.8 NA Plan Apo objective). Image stacks (2 μm/step) containing between 15–34 images were acquired of the up- and downstream regions of the OFT.

### 2.6. Confocal Image Analysis: Quantitative Immunofluorescence

**Figure 1:**
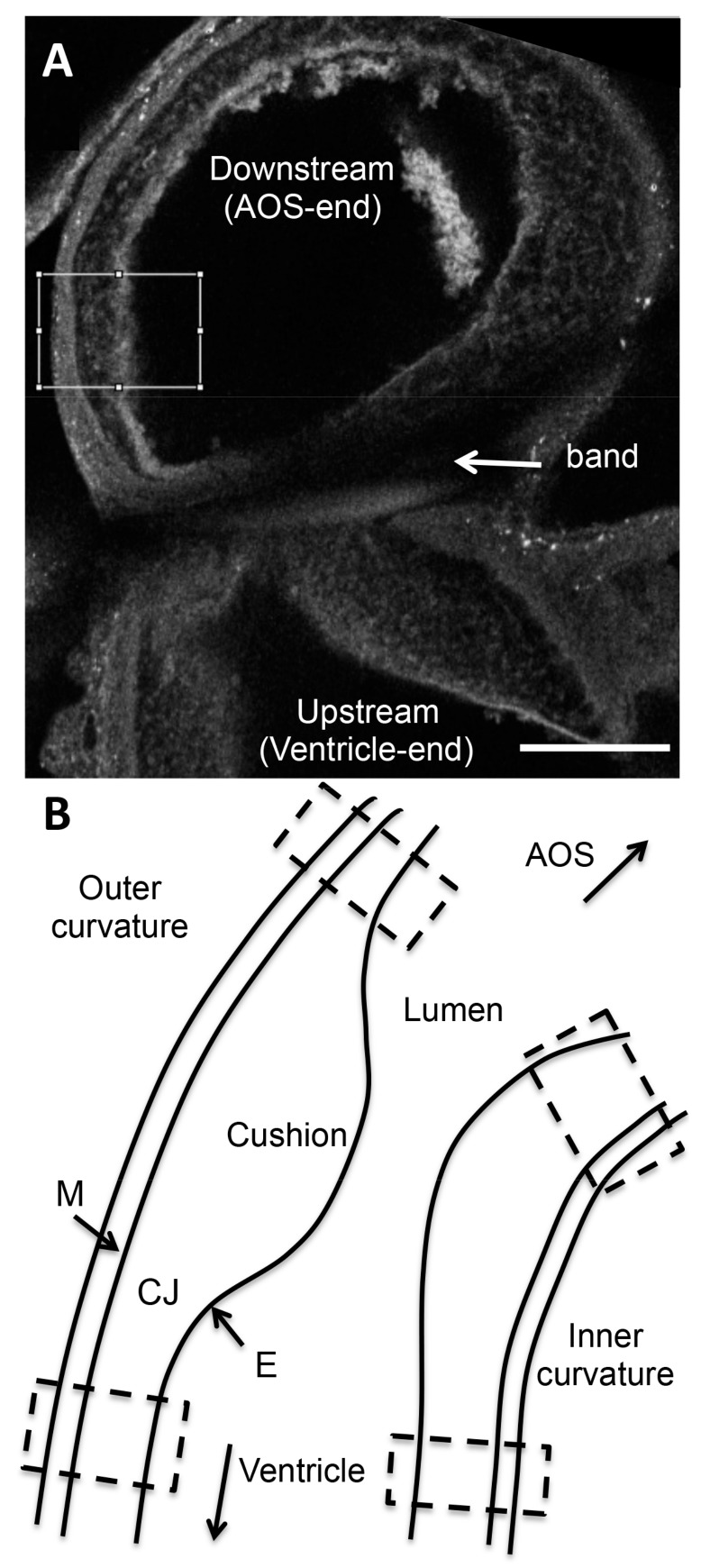
Intensity based confocal image analysis. (**A**) Example confocal image of the outflow tract (OFT) shown in grayscale. Intensities analyzed are grayscale pixel intensities (0 to 255 to represent black to white, with 255 defined as a saturation intensity). An example region of interest (ROI) selection (white box) is shown on this banded embryo immunostained for collagen VI. The box spans the three layers of the OFT wall (from left to right: myocardium, cardiac jelly, endocardium), downstream of the band location. Scale bar, 100 µm. (**B**) Schematic of the control OFT depicting the four regions in which confocal image intensity was analyzed (hatched boxes): downstream (closer to AOS) inner curvature, downstream outer curvature, upstream (closer to ventricle) inner curvature, upstream outer curvature. In banded embryos, the band is located in the mid-portion of the OFT, in between depicted hatched boxes. AOS, aortic sac; M, myocardium; CJ, cardiac jelly; E, endocardium.

Regional fluorescent intensity of confocal images was analyzed upstream and downstream of the band site (100–200 µm from the band or an equivalent location in shams), in both the inner and outer curvatures of the OFT (see Figure 1). For each location, 2–3 images from the z-stack that depicted the region of interest (ROI) were chosen for analysis. Each chosen image was rotated, enabling a rectangular (x-y) ROI to be selected in Image J (NIH) for further analysis. The horizontal length of the ROI (x-direction) spanned the three layers of the OFT wall (myocardium, cardiac jelly, and endocardium) and the ROI was 50–100 µm in height (vertical direction or y-direction). We then generated a plot of intensities along the ROI x-direction, by averaging pixel intensities in the y-direction (x-direction step size = 1.36 µm, corresponding to image pixel size). This plot depicted changes in confocal intensity along the OFT wall thickness, and therefore changes in protein content among wall layers.

Since OFT wall layers were apparent from confocal auto-fluorescence images, corresponding plots of intensity for the collagen of interest could be manually separated into segments matching the myocardium, cardiac jelly, and endocardium regions. Intensity data for each wall layer (segment) was then averaged over the thickness of the layer under consideration, and across the 2–3 images analyzed for that region, so that a single intensity value was obtained from each wall layer (myocardium, cardiac jelly, endocardium) for each sample position. Intensity values obtained in this manner were then corrected for background intensity by subtracting the average intensity value of a region of the OFT lumen (i.e., intensity with no tissue or blood cells present and therefore zero collagen deposition). Intensity values were then corrected for non-specific secondary antibody binding by subtracting intensity values calculated from negative control embryos. Steps taken to ensure reproducible relative intensity values among images, together with intensity correction steps, allowed for intensity comparisons among batches for the same collagen. Note, however, that intensity values are relative for each collagen, and thus obtained intensity values only represent a relative measure of concentration.

**Figure 2:**
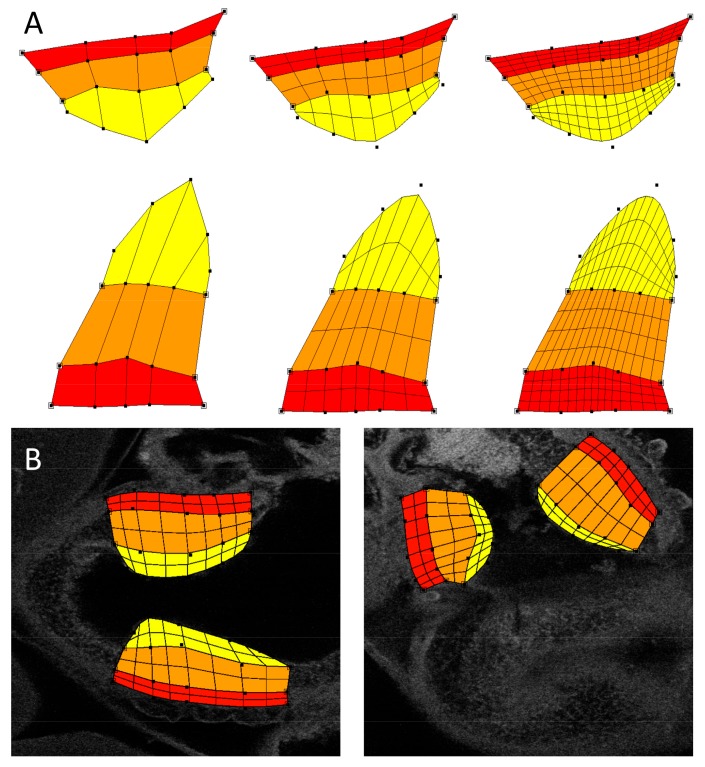
Atlas-based analysis of the outflow tract wall. Due to the geometrical complexities of the OFT cushions, an atlas-based intensity analysis was performed. Changes in cushion geometry will depend on biological variations among individuals as well as treatment, but also the chosen imaging plane (although efforts were made to choose corresponding planes from embryo to embryo). Subregions explicitly defined in the atlas are the myocardial wall (red), the outer region of the cushion (orange), and the cushion proper (yellow). (**A**) Example subdivision mesh for the OFT cushions, with different levels of spatial precision. The example was chosen to illustrate possible differences in cushion geometries from images; (**B**) The atlas was constructed using a control dataset as a template. The subdivision atlas is shown deformed to fit a control sham image (**left**) and an outflow tract banding (OTB) image (**right**). The atlas accommodates its geometry to that of the cushion geometry from confocal images.

A semi-automated flexible technique using atlas-based image registration was applied toward analyzing intensities in the OFT endocardial cushions (see Figure 2). This approach was adopted due to the variability in OFT cushion shapes compared to the cardiac wall layers outside of the cushion region described above. To analyze intensities specific to the cushions, we first constructed a 2D subdivision-based atlas utilizing the method previously described in [36]. Briefly, this approach applies the 2D subdivision mesh atlas construction tool described in [37] to delineate the cushions from confocal image sections. Cushion delineation is accomplished by denoting the vertices and edges of a quadrilateral mesh that fits to the cardiac layers in the cushion region of the OFT. Each quadrilateral is associated with a specific region (denoted by color in Figure 2A,B). The atlas was designed to explicitly distinguish featured regions of the OFT wall in the cushion region: myocardial wall, outer region of cushion (abutting the myocardial wall), and the inner OFT cushion or cushion proper (the region of the cardiac cushions that is closer to the lumen). The advantage of a subdivision mesh is that it can easily adjust its shape to match the heart and cushion morphology, but we can easily increase spatial resolution, which will ultimately affect intensity computations, by subdividing each quadrilateral (Figure 2A). A round of subdivision replaces each quadrilateral with four smaller quadrilaterals and refines the region boundaries into smoother curves. In this study, we report intensities at a level 1 subdivision, by averaging intensities inside each featured region.

For registering the subdivision atlas, we selected from each confocal dataset the 2D images that best captured the pair of OFT cushions. A small number of datasets (1–2 per collagen group) were excluded from analysis because they lacked a pair of OFT cushions within a single 2D image. To properly associate collagen localization from confocal images to the atlas, we used the atlas deformation tool [37] to adjust atlas shape to match the OFT cushion in the selected image region from each dataset. This involved manually moving the control points of the atlas to locations that allowed the explicit region boundaries of the atlas to properly match the corresponding boundaries in the confocal datasets. An example fitted atlas is shown in Figure 2B. With the atlas deformed to accurately overlay each image and featured region, each quadrilateral in the atlas was associated with the respective underlying intensity information in the image. To determine the average collagen density of a featured region, we calculated the mean intensity from pixel values inside a given atlas region. Intensity values were then corrected for secondary antibody effects as described above.

### 2.7. Quantitative RT-PCR

After hemodynamic intervention at HH18 (sham or OTB), embryos were re-incubated for about 24 h to HH24, and tissues from the OFT were collected for qRT-PCR. Prior to collection, in the OTB group, embryonic hearts were imaged immediately prior to banding and two hours after banding using optical coherence tomography (OCT) to assess band tightness (Equation (1)). To minimize sample-to-sample variations in OTB embryos due to different hemodynamic environments, only embryos with constrictions ranging from 30 to 40% band tightness at HH18 were used for qRT-PCR, as this range corresponds to maximum blood flow velocities and wall shear stresses among banded embryos [29].

To obtain sufficient quantities of RNA from the OFTs, ten HH24 OFTs were pooled per sample. Sample sizes were *n* = 5/group for collagens I, III, and VI, and *n* = 10/group for collagen XIV. The HH24 OFTs were excised over ice and stored in RNAlater^®^ (Life Technologies, Grand Island, NY, USA) at −20 °C until processing. Pooled OFTs were then homogenized in TRIzol^®^ reagent (Thermo Fisher Scientific, Eugene, OR, USA) and total RNA was isolated and reverse transcribed using the High-Capacity cDNA Reverse Transcription Kit (Thermo Fisher Scientific). Forward and reverse primer sequences employed are shown in Table 2. Quantitative PCR was performed in a 96-well Agilent Mx3005P QPCR System with Power SYBR Green (Thermo Fisher Scientific), and gene expression was quantified using the relative standard curve method. Exogenous standards (10^2^–10^−6^ ng/µL, in 10-fold dilutions) were prepared from amplified gene targets (PCR Master Mix 2X, Thermo Fisher Scientific) isolated from an agarose gel using the EZNA Gel Extraction Kit (Qiagen, Venlo, The Netherlands). Gene expression was calculated relative to the housekeeping genes ARBP and PGK-1, and normalized to the average control.

Due to large variations in the COL14A1 (collagen XIV) gene expression in the banded group, two batches of CON and OTB embryos (*n* = 5/group × 2 batches) were processed and gene expression quantified with qPCR. To account for variations in gene expression between batches, relative expression levels were normalized to the average control in each batch. For the other collagen genes, one batch of samples was employed (*n* = 5/group).

### 2.8. Statistics

Differences in blood flow velocities measured using Doppler ultrasound and gene expression using qPCR were assessed using a student’s *t*-test. Two-factor ANOVA was used to assess variance in signal intensity of wall layers obtained from confocal images. The factors accounted for in the ANOVA were treatment group (sham versus OTB) and immunostaining/imaging processing batch. Linear regression analysis was used to evaluate the relationship between confocal signal intensity and the degree of band constriction (at HH24). Statistical tests were performed using Graphpad Prism statistical software (GraphPad Software, Inc., La Jolla, CA, USA). Only *p*-values less than 0.05 were considered statistically significant.

## 3. Results

### 3.1. Measured OFT Blood Flow Velocity at HH24

**Figure 3:**
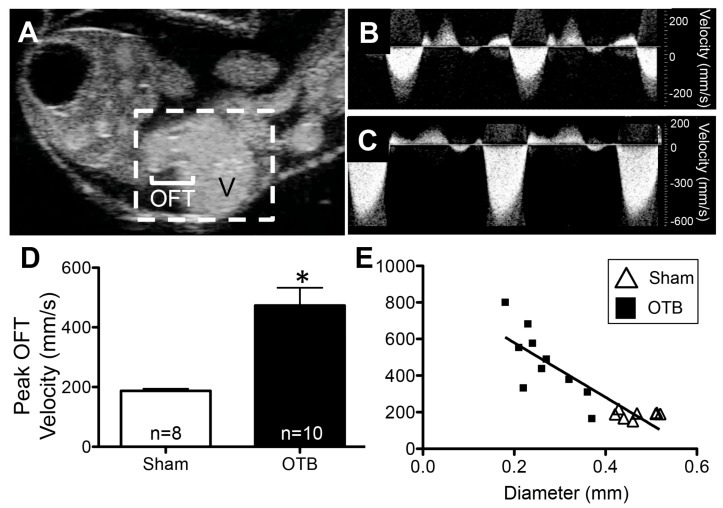
Ultrasound imaging at HH24 demonstrates increased blood flow velocity in the OTB group. (**A**) B-mode ultrasound image of the HH24 chicken heart (hatched white box). Blood can be seen pumping from the ventricle (V) through the outflow tract (OFT). In this still-frame image, the OFT lumen (white) is surrounded by darker OFT cushions, comprised of cardiac jelly. Example blood flow velocity waveforms in the OFT obtained from (**B**) control and (**C**) OTB embryos. (**D**) Peak OFT velocity was significantly larger in OTB embryos. * *p* < 0.05 via *t*-test. (**E**) Plot of peak OFT blood flow velocity versus maximum diameter of the OFT at the constriction site in banded embryos (squares) or equivalent location in sham controls (open triangles) demonstrates the relationship between band tightness and peak velocity increase.

High frequency ultrasound was used to image the OFT at HH24 (Figure 3A). Velocity waveforms acquired at the band location were similar in shape to those of sham controls (Figure 3B,C), but higher in magnitude as banding increased peak (maximum) OFT blood flow velocity by 2.5 fold (*p* < 0.0001, Figure 3D). Blood flow velocity through the banded OFT at HH24 was dependent on the degree of band constriction (20–60% band tightness, relative to average OFT diameter at an equivalent location in shams); a strong linear relationship was observed between peak velocity and OFT maximum diameter at the band site (Pearson’s correlation coefficient = −0.88, Figure 3E). In contrast, there was little variance in peak blood flow velocity within the control group despite a range of observed OFT diameters in sham embryos (0.42–0.57 mm) (Figure 3E).

### 3.2. Histological Section Comparisons

**Figure 4:**
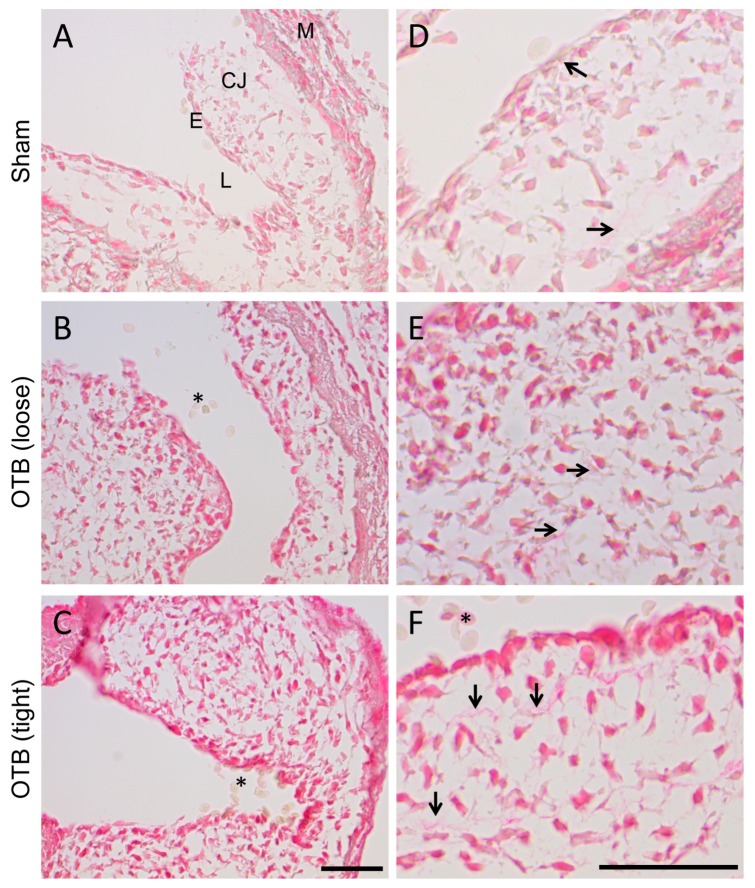
Picrosirius red staining for collagen suggests increased collagen deposition in the banded OFT. (**A**–**C**) Photomicrographs of the OFT wall in sham and banded embryos. Collagen containing tissue is stained in red, and appears to increase with progressive band tightness. M, myocardium; CJ, cardiac jelly; E, endocardium; L, lumen. Scale, 50 μm. (**D**–**F**) Photomicrographs of the cardiac jelly suggest increased collagen fibrils (arrows) in the tightly banded OFT. Red blood cells (*) in the lumen are the negative control. Scale, 50 μm.

Picrosirius red staining was performed to provide a general histological view of collagen and collagen deposition in the banded OFT in comparison to control hearts. Picrosirius red staining intensified with increasing band tightness (Figure 4A–C), suggesting increased collagen content. More intense cellular staining was observed in all layers of the OFT, indicating that cells are actively generating collagens. Additionally, extracellular staining of collagen fibrils increased in the cardiac jelly layer (mainly composed of glycosaminoglycans at HH24 and before), both in intensity of stain and the number of stained fibrils (Figure 4D–F). Changes were most pronounced in the tightest banded embryos.

### 3.3. Collagen I Distribution in the OFT

**Figure 5:**
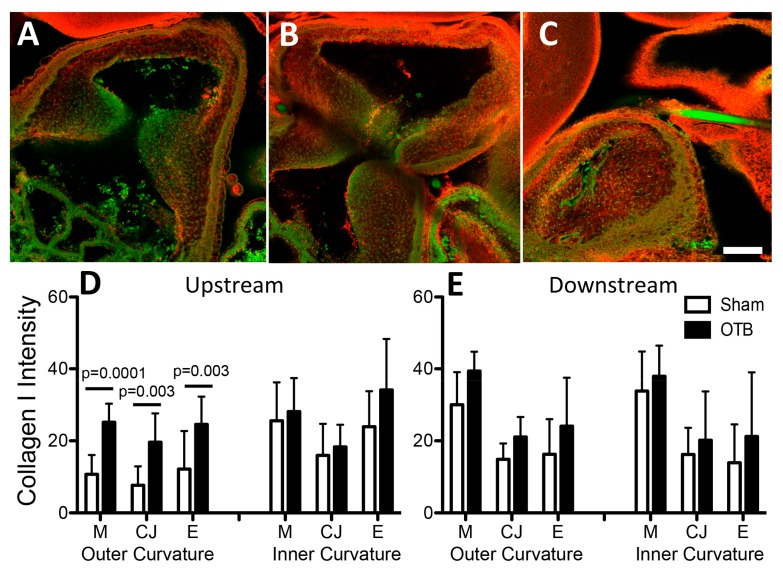
Collagen I deposition in the sham and banded OFT. Example confocal images are shown for (**A**) a sham control, (**B**) a moderately banded embryo, and (**C**) a tightly banded embryo. Collagen I deposition depicted in red, autofluorescence of the tissue depicted in green. Image intensity analysis results are depicted for (**D**) upstream of the band site (closer to the ventricle) and (**E**) downstream of the band site (closer to the aortic sac) in both the inner and outer curvatures of the OFT in each layer of the OFT wall (M, myocardium; CJ, cardiac jelly; E, endocardium). Sham data is shown in open bars, banded (OTB) data is shown in black bars. Intensity values represent pixel grayscale intensity. *p* values denote significance between sham and banded groups where appropriate. Data shown as mean ± SEM. Scale bar, 100 µm.

**Figure 6:**
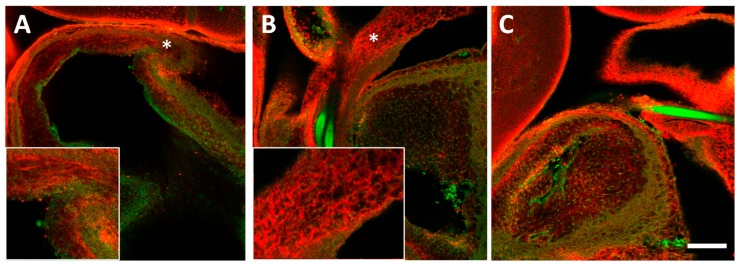
Collagen I fibrils (red) in the sham and banded OFT. (**A**) In sham embryos, several layers of collagen I fibrils were seen running longitudinally through the cardiac jelly layer of the OFT. The downstream region (*) is depicted in more detail in the inset. (**B**) In this representative tightly banded embryo (>30% constriction), increases in fibril organization and density were observed downstream (*) of the band site, highlighted in the inset region. (**C**) By contrast, in the same banded embryo, changes in fibril patterning were not observed upstream of the band (please note that Figure 6C is the same as Figure 5C). Tissue autofluorescence is overlayed in green on each image to better delineate the three layers of the OFT wall. Scale bar, 100 µm. Insets depict an area of about 125 × 125 µm^2^.

Collagen I was the most widely expressed collagen at the protein level; it was expressed in all layers of the HH24 OFT (Figure 5D,E) and closely mirrored Picrosirius red staining for total collagen deposition (Figure 5A). In the cardiac jelly, numerous collagen I fibrils running in parallel tracked the length of the OFT wall (Figure 6A, inset). Intensity analysis of immunofluorescent OFT images suggested that banding increased collagen I protein deposition by 2–2.5 fold in all layers of the OFT along the outer curvature upstream of the band site, i.e., close to the ventricle (Figure 5D). These increases were not significantly correlated with band tightness (data not shown), nor were any significant changes in expression observed downstream of the band. Interestingly, downstream of the band site, a change in fibril pattern/organization was observed in the most tightly banded embryos (band tightness >30%; Figure 6).

### 3.4. Collagen III Distribution in the OFT

**Figure 7:**
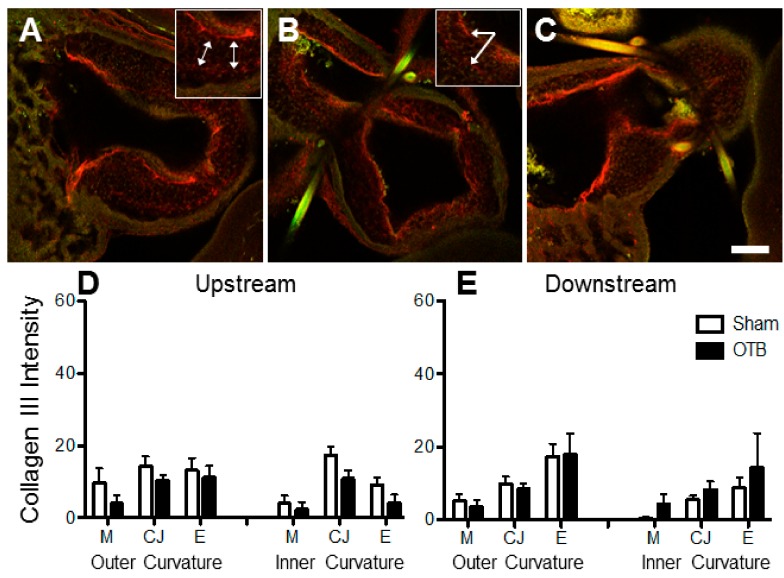
Collagen III deposition in the sham and banded OFT. Example confocal images are shown for (**A**) a sham control, (**B**) a moderately banded embryo, and (**C**) a tightly banded embryo. Collagen III deposition depicted in red, innate autofluorescence of the tissue depicted in green. Highly oriented fibrils (double sided arrows) are highlighted in the inset panel of (**A**) and in the inset panel of (**B**) (arrows). Image intensity analysis results are depicted for (**D**) upstream of the band site (closer to the ventricle) and (**E**) downstream of the band site in both the inner and outer curvatures of the OFT in each layer of the OFT wall (M, myocardium; CJ, cardiac jelly; E, endocardium). Sham data is shown in open bars, banded (OTB) data is shown in black bars. Intensity values represent pixel grayscale intensity. *p* values are not shown as collagen III deposition was not significantly upregulated in any region of the banded OFT. Data shown as mean ± SEM. Scale bar, 100 µm.

Collagen III seemed unchanged with banding (Figure 7). After OFT immunofluorescence, collagen III protein was visible in the cardiac jelly and endothelial layers of the OFT, where prominent fibrils (1 to 2) lined the OFT wall (e.g., Figure 7B). Less prominent fibrils in the cardiac jelly dispersed radially towards the myocardium and endocardium (e.g., Figure 7A). Quantitative image analysis detected minimal traces of collagen III in the myocardium that were comparable to image noise level; only collagen III deposition in the cardiac jelly and endocardium were statistically different to zero (95% confidence intervals, Figure 7D,E).

### 3.5. Collagen VI Distribution in the OFT

**Figure 8:**
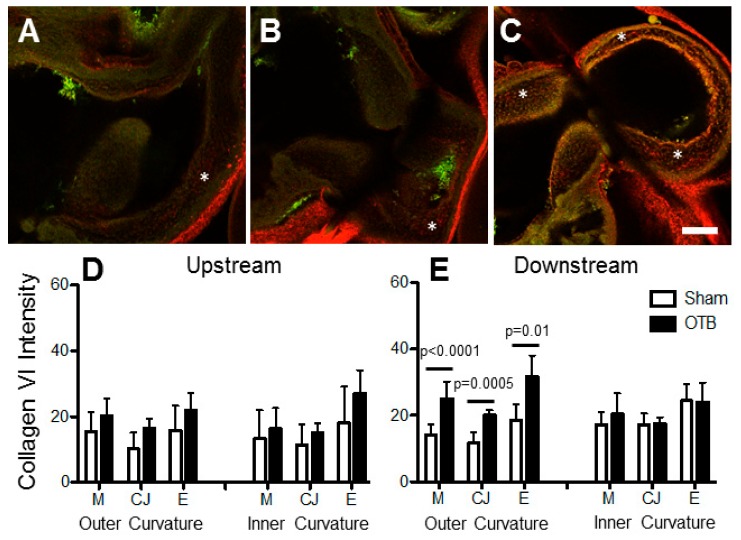
Collagen VI deposition in the sham and banded OFT. Example confocal images are shown for (**A**) a sham control, (**B**) a moderately banded embryo, and (**C**) a tightly banded embryo. Collagen VI deposition depicted in red, innate autofluorescence of the tissue depicted in green. Punctate expression of collagen VI in the cardiac jelly is highlighted by (*). Image intensity analysis results are depicted for (**D**) upstream of the band site (closer to the ventricle) and (**E**) downstream of the band site in both the inner and outer curvatures of the OFT in each layer of the OFT wall (M, myocardium; CJ, cardiac jelly; E, endocardium). Sham data is shown in open bars, banded (OTB) data is shown in black bars. Intensity values represent pixel grayscale intensity. *p* values denote significance between sham and banded groups where appropriate. Data shown as mean ± SEM. Scale bar, 100 µm.

Collagen VI expression was present at low levels throughout the HH24 OFT (Figure 8). Its deposition was most obvious in the cardiac jelly layer, where it was observed in patches, including in the OFT cushions. Unlike collagens I and III, collagen VI is not a fibril collagen, and fibril-like structures were not observed. Collagen VI was strongly expressed in the pericardial sac lining the OFT (Figure 8A,C). After image intensity analysis, collagen VI protein deposition seemed elevated by 1.7 fold in the outer curvature downstream of the band site in each of the layers of the OFT (Figure 8E).

### 3.6. Collagen XIV Distribution in the OFT

**Figure 9:**
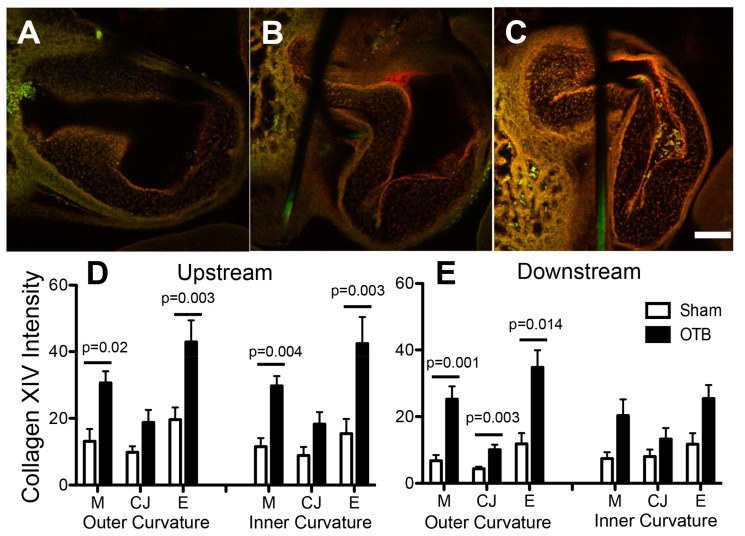
Collagen XIV deposition in the sham and banded OFT. Example confocal images are shown for (**A**) a sham control, (**B**) a moderately banded embryo, and (**C**) a tightly banded embryo. Collagen XIV deposition depicted in red, innate autofluorescence of the tissue depicted in green. Overlapping expression appears yellow-orange. Image intensity analysis results are depicted for (**D**) upstream of the band site (closer to the ventricle) and (**E**) downstream of the band site in both the inner and outer curvatures of the OFT in each layer of the OFT wall (M, myocardium; CJ, cardiac jelly; E, endocardium). Sham data is shown in open bars, banded (OTB) data is shown in black bars. Intensity values represent pixel grayscale intensity. *p* values denote significance between sham and banded groups where appropriate. Data shown as mean ± SEM. Scale bar, 100 µm.

**Figure 10:**
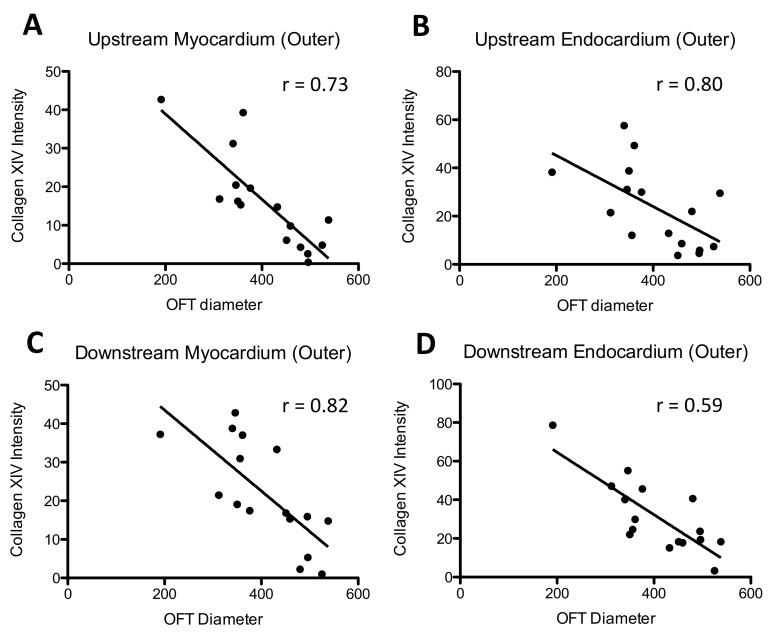
Correlation between collagen XIV intensity and OFT diameter. Intensity values represent pixel grayscale intensity. Intensity was computed from image analysis of collagen XIV immunofluorescent embryos in the regions of the heart OFT. OFT diameter is related to band tightness, with larger diameters corresponding to control (unbanded) samples and band tightness increasing as OFT diameter gets smaller. OFT diameter was estimated from confocal images of the HH24 immunofluorescent embryos. Regions plotted are the outer curvature, upstream of the band in (**A**) myocardium; and (**B**) endocardium; and the outer curvature, downstream of the band in (**C**) myocardium; and (**D**) endocardium.

The most dramatic increases resulting from banding were observed in collagen XIV expression, which increased both upstream and downstream of the band site, as well as in the OFT cushions, in striking contrast to its minimal to absent expression in sham controls (Figure 9). Intensity image analysis indicated that upstream of the band site, collagen XIV protein deposition was increased in the myocardial and endocardial layers of both the inner and outer curvatures of the OFT, but not in the cardiac jelly (Figure 9D). Downstream of the band, no significant changes were observed along the inner curvature of the OFT. In the outer curvature, however, elevations in collagen XIV were observed in the myocardium (*p* = 0.001), cardiac jelly (*p* = 0.003), and endocardium (*p* = 0.01). A significant linear correlation was observed between the degree of expression (intensity levels) and band tightness in every region where collagen XIV was upregulated except for the OFT cushions (Table 3; Figure 10), suggesting that collagen XIV is highly sensitive to hemodynamic conditions.

### 3.7. OFT Atlas-Based Cushion Analysis of Collagen Distribution in the Cushions

**Figure 11:**
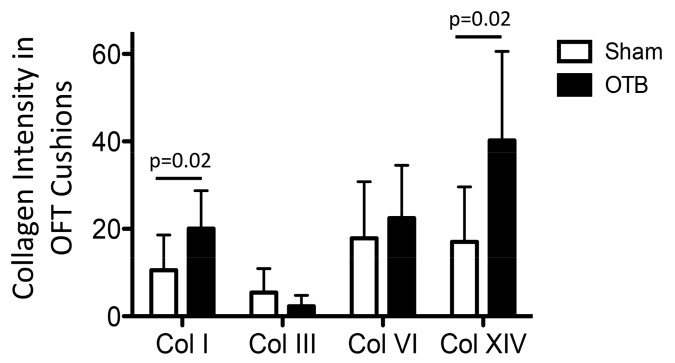
Deposition of collagens I, III, VI, and XIV in the endocardial cushions of the sham and banded OFT. Intensity analysis results from atlas-based registration are shown for sham controls (open bars) and banded groups (OTB, black bars). The analysis was done based on intensities in the cushion proper region, closed to the endocardium (see Figure 2). Intensity values represent pixel grayscale intensity. *p* values denote significance between sham and banded groups where appropriate. Data shown as mean ± SEM. Please note that intensity measurements are relative to each specific collagen analyzed and do not represent abundance of the different collagen types in the heart wall (e.g., collagen I is much more abundant than collagen XIV, even though intensity values seem to suggest the opposite).

To analyze collagen intensity in the OFT endocardial cushions, we used an atlas-based analysis that allowed us to account for the varying cushion geometry (Figure 11). Atlas-based analysis results suggest that banding increased collagen I and collagen XIV protein deposition (by 2-fold) in the OFT cushions (*p* = 0.02).

### 3.8. Collagen Gene Expression in the OFT

**Figure 12:**
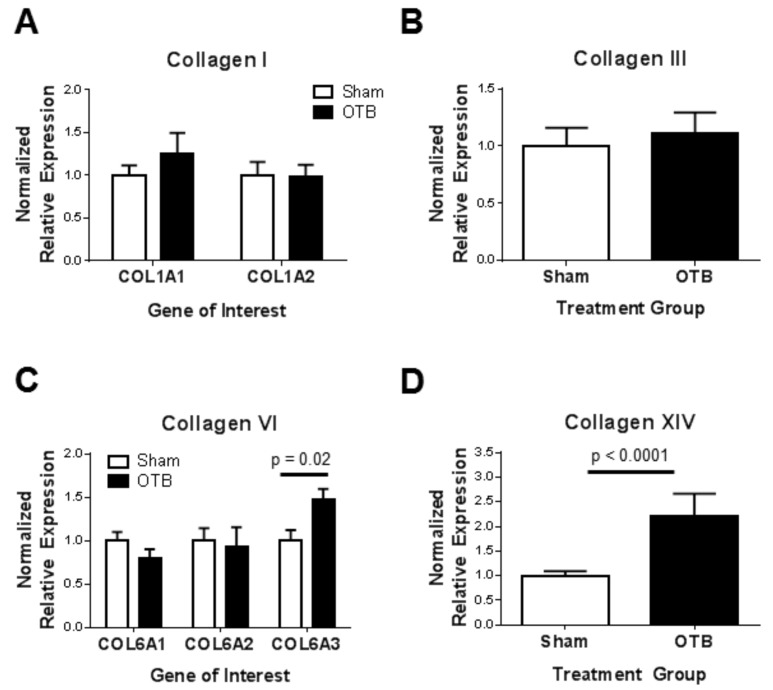
Collagen gene expression in the sham and banded OFT. Quantitative real-time PCR was performed on pools of 10 banded and sham OFT (*n* = 5 for collagens I, III, and VI; *n* = 10 for collagen XIV) after 24 h of hemodynamic intervention (at HH24). Data are expressed as the normalized expression relative to the housekeeping gene, PGK-1. Comparisons of collagen transcript levels between banded and control OFTs are shown for (**A**) Collagen I; (**B**) Collagen III; (**C**) Collagen VI; (**D**) Collagen XIV. Data shown as mean ± SEM.

We used qRT-PCR to determine RNA levels of collagen genes and determine whether they have changed in response to the banding procedure (Figure 12). While expression data was normalized to the expression of two housekeeping genes, ARBP and PGK-1, results rendered by these normalizations were similar. Although a slight increase in COL1A1 gene expression was observed, neither collagen I transcript (COL1A1, COL1A2) changed significantly with banding. Similarly, COL3A1 gene expression was not significantly different between banded and control OFT samples. However, gene expression of collagen VI (COL6A3) was increased by 1.5-fold, while expression of the remaining collagen VI genes (COL6A1, COL6A2) did not change significantly in the OTB group. Collagen XIV gene transcript level showed little variation in the sham control group, but was highly variable in the banded group despite the narrow range of band tightness analyzed by qPCR. Nonetheless, COL14A1 expression increased significantly by 2.2-fold after banding.

For comparative purposes, a summary of the findings is shown in Table 4.

## 4. Discussion

Alterations in blood flow at early embryonic stages can lead to congenital heart defects [1,3,4,5,6,7,40], yet early detrimental embryonic tissue remodeling in response to perturbed hemodynamic conditions is not well understood. In this study, we characterized changes in collagens I, III, VI and XIV in response to increased hemodynamic load, achieved by a banding procedure in the embryonic heart OFT during tubular heart stages (HH18 to HH24). Prior work has shown that blood pressure increases throughout the OFT after banding, with blood pressure increasing nonlinearly with increased band tightness [30]. Cardiac blood pressure remains elevated (with respect to controls) after 24 h of banding at HH24. Blood flow velocity (and thus wall shear stress) also increases in the banded region as a function of band tightness both immediately (~2 h) after banding [29] and after 24 h of intervention (Figure 3). Wall shear stress varies throughout the OFT endocardial surface (in space and time) as a function of band tightness, and is in general elevated in banded versus control hearts [41,42], especially on the cushion endocardium. Moreover, computational fluid dynamics modeling of the OFT revealed that wall shear stress is, in general, higher in the inner than in the outer curvature of the OFT [41,42]. Optical coherence tomography imaging further reveals that longitudinal stretch during the cardiac cycle is elevated in the outer curvature OFT wall in comparison to the inner curvature OFT wall. Thus, location-specific changes in collagen content observed in this study likely reflect local shear and/or wall stress patterns in the banded OFT.

It is worth noting that, while both protein and gene expression levels were quantified in this study, these quantifications are somewhat complementary. An obvious difference is that while protein expression, quantified with immunofluorescence, captures localized variations in collagen content; gene expression, quantified from samples that pooled together 10 whole OFTs, can only capture global changes, missing local variations. Moreover, transcriptional and post-transcriptional regulation may independently affect the tissue adaptation to blood flow. It is worth mentioning, in addition, that while quantitative immunofluorescence has been recently used in studies (e.g., [34,35]), quantification of immunofluorescence remains a highly controversial subject. Many experts in the field do not believe that accurate quantifications of relative protein contents could be extracted from immunofluorescence in the way done in this paper. Rather, they argue that immunofluorescence can only be used to extract protein expression patterns. As shown in other studies, we believe that quantification is possible, and relatively accurate, providing that certain precautions are taken: (1) Samples need to be prepared and immunostained in exactly the same way, making sure that timing, concentrations, etc., match exactly from sample to sample in a batch that contains both control and intervention samples; (2) Images need to be acquired in exactly the same way, in one session, without altering acquisition settings or parameters among the same protein samples in the batch. These steps will ensure consistency. Of course, one can argue that other factors, such as potential differential photobleaching of samples, can still occur. While we cannot control all factors, we believe that the steps taken in this work to ensure image consistency (listed above and in more detail in the methods) are enough to compare relative amounts of collagen contents among embryonic hearts. In light of controversies, however, the reader should be aware of caveats in the interpretation of our results.

This study reveals that collagen XIV, minimally present in the sham OFT, was by far the most sensitive collagen to hemodynamic load, with increases shown at the protein and RNA levels. Collagen XIV deposition in the embryonic heart has previously been localized to the endocardial cushions, cardiac muscle, and basement membrane, albeit at much later stages of development when mechanical stress is higher [22,43,44]. Indeed, collagen XIV is often localized to areas of high mechanical stress in embryonic tissue such as the cardiac muscle, tendon, and ligament/bone junction [43,45]. Prior work has suggested a role for collagen XIV in determining tissue properties in mechanically stressed tissues during development [23]. Hearts of mice lacking the COL14A1 gene have reduced ejection fraction and, following pressure overload, develop exacerbated thickening of the left ventricular wall [22], suggestive of a detrimental effect on wall structure and function. Tendons of collagen XIV null mice have reduced strength and stiffness [23], and heterozygotes, expressing an intermediate amount of collagen XIV, exhibit intermediate mechanical properties in the tendon. Here, after banding the heart at HH18, blood pressure and wall shear rate, and thus cardiac tissue stress, significantly increases in the chick heart OFT in a band-tightness dependent manner [29,30]. The significant correlation we observed between collagen XIV deposition and band tightness suggests that collagen XIV production is highly sensitive to hemodynamic conditions at this developmental stage, and may be an important player in modulating load-induced cardiac tissue remodeling.

Increased collagen XIV deposition could also be at least partially responsible for the increased organization observed in collagen I fibrils. In the most tightly banded embryos, increased collagen I fibril organization was observed downstream of the band in the cardiac jelly, in a region where we also showed collagen XIV deposition to be dramatically upregulated. Increased fibril organization was not observed in regions of the banded OFT where collagen XIV deposition was unchanged. Several studies have linked collagen XIV, which interacts with collagen I through a collagen I binding domain [46], to collagen I fibril organization during the embryonic period, and suggested that the role of collagen XIV in regulation of fibrillogenesis is hemodynamically regulated and transient, occurring mostly during early developmental stages. In zebrafish embryos, for example, collagen XIV expression peaks early in development, and is thought to regulate collagen I fibril assembly in the developing tendon during the longitudinal fibril growth, but not during the fibril thickening that occurs at more mature stages [47,48]. In the embryonic OFT, we found that the deposition pattern of collagen XIV closely mirrored that of collagen I. Co-localization of these collagens in embryonic tissue, however, is not limited to the heart OFT: in the chicken embryo, localized gene and protein expression of collagen XIV mirrors that of collagen I in virtually every collagen I containing embryonic tissue [43]. Together, these findings suggest that collagen XIV’s role in mediating collagen I fibrillogenesis is unique to the embryo and neonate, possibly explaining why collagen XIV expression in the heart is higher during development and the postnatal period than in adult tissue [22] and why increased collagen XIV has not been found in the numerous studies demonstrating increased collagen I deposition in the overloaded adult heart [49].

Unlike in the mature heart when increased blood pressure leads to increased collagen I and III deposition [16,49], our study shows no overall increase in the abundance of collagens I and III with banding (and thus increased blood pressure). Although elevated collagen I deposition was observed in the OFT cushions and along the outer curvature upstream of the band, and increased fibril organization was visualized, overall collagen I gene expression in the OFT tissue does not significantly change, implying perhaps a change in the deposition pattern or distribution of collagen I rather than an overall increase in abundance. This response to overload in the OFT is somewhat different to the load-induced increases in collagen I deposition in adult heart tissue [16,49], and in the embryonic dorsal aorta [20]. In both those cases, collagen I deposition increases with increased blood pressure. Our results, suggesting a change in the distribution of collagen I together with increased organization, would imply localized changes in stiffness [50] that may be responsible for the later formation of cardiac defects in banded hearts [6,7].

Whereas later in development, collagen III is essential for collagen I fibrillogenesis in the embryonic heart, we did not observe an increase in collagen III anywhere in the OFT, including in regions where collagen I fibrillogenesis appeared to be upregulated. Similarly, collagen III levels in the newborn rat ventricle do not increase after 14 days of aortic banding [51]. Our data show that the pattern of collagen III deposition was remarkably conserved across all embryos observed, suggesting that collagen III is not an early ‘player’ in the hemodynamic induced remodeling of the embryonic OFT. Interestingly, pressure-overload in the newborn rabbit heart does not alter ventricular collagen III content, yet six weeks later a similar overload leads to a collagen III increase [52]. Further, collagen III has been shown to increase in the overloaded embryonic chicken vasculature after 48 h of hemodynamic intervention [20]. It is thus possible that a hemodynamic intervention later in development or longer periods of banding would result in increased deposition perhaps of both collagen I and III. Developing heart tissue may have a unique response to overload, different to that of more mature cardiac tissues.

Collagen VI content seems to increase in embryonic heart tissue over development [53], coincident with increases in wall shear stresses and blood pressure over developmental stages. In banded embryos, COL6A3 gene expression was elevated in the OFT. Immunofluorescent images, however, suggest an increase in collagen VI deposition downstream of the band in the outer curvature (in the region of increased collagen I fibril organization). In embryonic mice, COL6A3 gene expression directly precedes the presence of collagen VI, suggesting the expression of the COL6A3 gene, rather than COL6A1 or COL6A2, regulates the formation and secretion of collagen VI trimmers and matrix deposition during development [54]. However, studies in the adult heart suggest that excess myocardial collagen VI content is detrimental for heart function, and collagen VI is also associated with prevalence of cardiac defects in down syndrome patients [25]. In human patients with hypertrophic cardiomyopathy, myocardial collagen VI content inversely correlates with ejection fraction [55]. Additionally, post myocardial infarction cardiac function and remodeling is improved in mice lacking the COL6A1 gene relative to wild type controls [56]. Thus, prior work demonstrated a role for collagen VI in pathological cardiac remodeling of the adult heart. The elevated collagen VI deposition observed after banding in the current study may contribute to detrimental remodeling of the embryonic heart wall, or may be a natural mechanism in response to elevated hemodynamic load in the heart. 

## 5. Conclusions

Hemodynamic overload during early heart development alters OFT collagen distribution, which may lead to a detrimental tissue remodeling and the formation of cardiac defects. The observed localized increases in collagen I, VI and XIV deposition, with increasing degree of constriction, suggest that these collagens play a key role in structural adaptation to increased hemodynamic load during this early embryonic period. Of interest, we found co-localization of collagens I and XIV in the embryonic OFT tissue, and increased organization of collagen I fibers in regions in which collagen XIV deposition was significantly increased. The location-specific increased deposition likely reflect local wall shear and wall stress patterns in the constricted OFT. In fact, the outer curvature is exposed to elevated cyclic stretch while the inner curvature is exposed to elevated wall shear stress in the same embryonic heart, with hemodynamic stimuli increasing in banded embryos. Changes in collagen content could be due to both transcriptional and post-transcriptional events, perhaps suggesting a very dynamic early adaptation to altered blood flow conditions that needs to be further investigated. In the future, these data will contribute to a better understanding of early changes in cardiac wall adaptation to hemodynamic conditions and detrimental cardiac remodeling that underlie heart defects and adult onset cardiovascular disease.

## Figures and Tables

**Table 1:** Summary of characteristics of collagen proteins studied.

Collagen	Type	Triple Helix Chains	Genes
**I**	Fibril	α1(I), α2(I)	*COL1A1*, *COL1A2*
**III**	Fibril	α1(III)	*COL3A1*
**VI**	FACIT ^1^	α1(VI), α2(VI), α3(VI)	*COL6A1*, *COL6A2*, *COL6A3*
**XIV**	FACIT ^1^	α1(XIV)	*COL14A1*

^1^ FACIT: Fibril-associated collagen with interrupted triple helix.

**Table 2:** Forward and reverse primer sequences used for collagens in the chick OFT. The ARBP and PGK-1 primer sequences were obtained from [38,39], respectively. Other primer sequences were designed by our group using Primer Designer software (San Diego State University Biological Workbench, San Diego, CA, USA; and Northwestern University Oligo Calc, Chicago, IL, USA).

Gene	Forward Sequence	Reverse Sequence
**COL1A1**	TGC TGT TGA TAG CAG CGA CT	GTG TCC TCG CAG ATC ACC TC
**COL1A2**	TGG TAG GGT TGG GCC AAT CG	GCA CCT TGG TTG CCA GTG AC
**COL3A1**	CTG GTT TCC GTG GCT TAC CC	GCC AGG CTT CCC ATC ACT TC
**COL6A1**	TCG CCT ATT CCA AGG GAA AC	CTG CGA TGT GAT GAC CTA CG
**COL6A2**	CTC GGC GCA ATG CTG AAC TC	ATG GCA GCC CGA TCT CCA AG
**COL6A3**	CTC CAT CAA CCT GGG AAG AG	GGC CAC CTT TGT AGA TCA CC
**COL14A1**	GTC CCG AGG AAG CGA TAA TG	AGG AGC AGA CAA GGG TAA CG
**ARBP**	CCT GTG ATG TGA CTG TGC	ACT TTG TCT CCG GTC TTA ATC
**PGK-1**	AAA GTT CAG GAT AAG ATC CAG CTG	GCC ATC AGG TCC TTG ACA AT

**Table 3:** Collagen XIV image signal intensity correlates with band tightness. Correlation between immunostained image intensity in each cardiac layer and OFT diameter, which is related to band tightness, quantified by r and *p*-value.

Position Relative to Band	Wall Curvature	Wall Layer	r	*p*-Value
Downstream	Outer	Myocardium	0.82	**<0.001**
		Cardiac Jelly	0.71	**0.002**
		Endocardium	0.59	**0.0164**
	Inner	Myocardium	0.46	NS
		Cardiac Jelly	0.27	NS
		Endocardium	0.23	NS
Upstream	Outer	Myocardium	0.73	**0.001**
		Cardiac Jelly	0.45	NS
		Endocardium	0.80	**<0.001**
	Inner	Myocardium	0.73	**0.002**
		Cardiac Jelly	0.27	NS
		Endocardium	0.72	**0.002**

r, Pearson’s correlation coefficient; *p*-value determined via linear regression analysis, statistically significant values are shown in bold.

**Table 4:** Summary of localized increases in collagen deposition throughout the banded OFT. Arrows indicate a significant upregulation in collagen deposition in the banded OFT (*p* < 0.05). M: myocardium; CJ: cardiac jelly; E: endocardium; OC: outer region of cushion (adjacent to the myocardium); CP: cushion proper (adjacent to the endocardium).

Type	Upstream						Downstream								
	Outer Curvature			Inner Curvature			Outer Curvature			Inner Curvature			Cushions		Whole OFT
	M	CJ	E	M	CJ	E	M	CJ	E	M	CJ	E	OC	CP	qPCR
I	↑	↑	↑				↑Fibril organization							↑	
III															
VI							↑	↑	↑						↑COL6A3
XIV	↑		↑	↑		↑	↑	↑	↑					↑	↑
